# Infliximab-induced Depression and Suicidal Behavior in Adolescent with Crohn’s Disease: Case Report and Review of Literature

**DOI:** 10.1097/pq9.0000000000000229

**Published:** 2019-11-18

**Authors:** Michelle Shayowitz, Moshe Bressler, Alison P. Ricardo, Eugene Grudnikoff

**Affiliations:** * Department of Psychiatry, Elmhurst Hospital, New York City, N.Y.; † New York Institute of Technology College of Osteopathic Medicine, Old Westbury, NY; ‡ Donald and Barbara Zucker School of Medicine at Hofstra/Northwell, NY

## Abstract

Infliximab, an anti-inflammatory agent, is used to treat various autoimmune disorders. There are at least 3 reports of severe psychiatric adverse effects of the drug, including suicidal behaviors in adults and psychosis in adult and adolescent patients. We report a case of an adolescent who developed depression and suicidal behaviors shortly after beginning infliximab. Although there have been reports of adolescents developing acute psychosis shortly after starting infliximab, this is, to our knowledge, the first report of adolescent suicidal behavior in the setting of infliximab treatment.

**Methods:**

We describe a patient’s presentation and clinical course, as well as existing reports of adverse psychiatric effects of infliximab.

**Results:**

A 16-year-old male with a 2-year history of disabling symptoms and complications of Crohn’s disease was initiated on a trial of infliximab. Within days of the first infliximab infusion, he experienced symptoms of depression, which intensified over weeks and resulted in a serious suicide attempt. The patient was treated with fluoxetine, melatonin, and psychotherapy, which effectively managed his infliximab-induced depressive disorder with suicidal thoughts and behaviors. Posttreatment, he tolerated additional infliximab infusions without the recurrence of psychiatric symptoms.

**Conclusions:**

Treatment with infliximab may rarely and suddenly cause severe and potentially life-threatening psychiatric symptoms. Therefore, youth with chronic illnesses considered for infliximab treatment should be screened for preexisting, as well as for a family history of, psychiatric disorders and suicidal behavior.

## INTRODUCTION

Infliximab is a tumor necrosis factor-alpha (TNF-α) inhibitor commonly used in the treatment of Crohn’s disease.^1^ There are several documented adverse effects including an increased risk of opportunistic infections, malignancy, and neurodegenerative diseases.^2^ Additionally, several reports exist describing the new-onset of psychiatric symptoms linked to infliximab treatment, such as suicidal behaviors in adults and elderly patients, as well as psychosis in an adolescent.^2–5^ Additionally, another TNF-α inhibitor, etanercept, has also been implicated in causing a psychotic reaction.^6^ Here, we present a case of an adolescent male who developed acute onset depression with resultant suicidality after his fourth infusion of infliximab. To our knowledge, this is the first report of such a case in an adolescent.

## CASE PRESENTATION

**Fig. 1.**
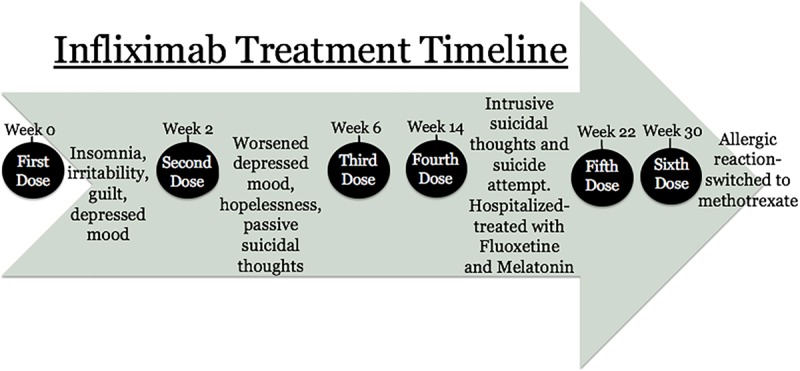
Infliximab treatment timeline, including adverse effects and interventions.

The patient is a 16-year-old White male with a history of Crohn’s disease diagnosed in 2014. When first diagnosed, he experienced bouts of joint pain, abdominal pain, and diarrhea, resulting in the loss of 14 kg in 6 months. Further complications included buttock abscesses and anal fissures, requiring several surgeries. In May of 2016, he began treatment with infliximab (5 mg/kg) and received subsequent infusions at weeks 2 and 6 (Fig. 1).

Within days of initiating treatment, the patient had significant improvement in his gastrointestinal symptoms. However, within a week of the first infusion, he developed persistent insomnia, periods of increasing irritability, depressed mood, and guilt. During the 2 months following his second infusion, he experienced further worsening of depressed mood, hopelessness, and the onset of passive suicidal thoughts. He received a third infusion at week 8 and a maintenance infusion at week 14. Within 24 hours of his fourth infusion, his depressive symptoms significantly worsened, and his suicidal thoughts became intrusive. The patient subsequently attempted to hang himself with a belt, but the belt quickly broke. After a period of altered level of consciousness, he was taken to the emergency room and admitted to the psychiatric unit. He reported that before beginning infliximab, he had felt stressed, anxious, and had brief periods of depressed mood due to the severe symptoms of his Crohn’s disease. However, he did not experience disabling depressive syndrome, suicidal thoughts, or ever made a prior suicide attempt. Despite the resolution of his gastrointestinal symptoms, his new-onset suicidal thoughts became intermittently intrusive and ego-dystonic; he experienced clear intent to die without any ambivalence at the time of the attempt.

Additionally, he reported a period of frequent cannabis use before treatment with infliximab, as he tried to alleviate the anxiety and distress related to his illness. He denied any previous episodes of mania or psychosis or a history of trauma. His mother reported an increase in agitation and episodes of mood swings during the preceding weeks of his suicide attempt. Family psychiatric history included bipolar depression in his father and depression with suicidal behaviors in both of his brothers. Upon admission, his thyroid panel, complete blood count, complete metabolic panel, and urine toxicology were all within normal limits.

The patient was diagnosed with the infliximab-induced depressive disorder; we categorized his symptoms of depression and anxiety before the start of infliximab as adjustment disorder with mixed anxiety and depressed mood. During his hospitalization, fluoxetine 10 mg, which was started in the emergency room, was continued and increased to 20 mg before discharge. He received melatonin 6 mg for insomnia. At discharge, his depressive symptoms had diminished significantly, and he no longer experienced suicidal thoughts. He was referred to follow-up with an outpatient therapist and psychiatrist. His psychiatrist recommended that if symptoms of depression persist, he should begin treatment with risperidone before his next infusion of infliximab to prevent the recurrence of acute intrusive suicidal thoughts.

After discharge, he continued taking fluoxetine with a sustained response; he was generally in a good mood and had no suicidal thoughts. He tolerated his fifth infliximab infusion without pretreatment with risperidone. At his sixth treatment, he developed an allergic reaction with symptoms of anxiety and shortness of breath. At the time of the latest contact with the family, the patient had been switched to methotrexate due to the ongoing complications of infliximab therapy and had no recurrent psychiatric illness since his discharge.

## DISCUSSION

Although the use of TNF-α inhibitors has been associated with several adverse effects, there is little known about infliximab’s psychiatric side effects.^2^ Recognizing the impact of the drug on a patient’s psychiatric status is difficult due to the high rate of comorbidity of psychiatric conditions secondary to the medical conditions for which TNF-α inhibitors are originally prescribed. TNF-α, along with other pro-inflammatory cytokines, has been identified as a factor in the pathogenesis of various neuropsychiatric conditions.^6^ A genetic association study found that TNF-α is one of the most fundamental shared genes involved in major depression and suicidal behavior.^7^ When examining postmortem brains of suicide victims, regardless of the psychiatric condition, there was a 2.5-fold greater expression of TNF-α in the dorsolateral prefrontal cortices of individuals who died of suicide compared with controls.^8^ One way in which TNF-α induces these behavioral changes is its effect on the metabolism of neurotransmitters involved in depression and suicidality in both the limbic system and basal ganglia. Also, pro-inflammatory cytokines have a profound stimulatory effect on hypothalamic–pituitary axis hormones and corticotropin-releasing hormone, which are pathways that also contribute to the development of depression. However, animal studies have also shown neuroprotective effects of TNF-α at different regions of the brain.^6^ Therefore, the extent to which the reduction of endogenous TNF-α contributes to neuropsychiatric effects is not clear.

Several case reports exist that display a pattern of rapid onset neuropsychiatric symptoms just months after beginning TNF-α inhibitor therapy despite an improvement of the medical condition, which may be indicative of shared pathogenesis.^3,4^ Remarkably, in each of these cases, additional risk factors for depression and suicide exist, such as a family history or concurrent psychiatric and medical illness, which likely contributed to their susceptibilities. Similarities across these case reports implicate TNF-α inhibitor action and strengthen the association between infliximab treatment and the onset of psychiatric adverse effects, but further investigation is warranted for evidence of a causative relationship.

The known association between TNF-α inhibitor treatment and the onset of psychiatric symptoms should result in the proper assessment of patients for psychiatric conditions before beginning therapy. In fact, due to the higher rates of depression and anxiety seen in patients with inflammatory bowel disease, the American College of Gastroenterology guidelines recommend screening all patients with inflammatory bowel disease for psychiatric illnesses.^9^ The incidence of infliximab-induced depression is likely underreported, and thus specific attention must be given to detect its occurrence.^10^

In this case, in addition to being at increased risk for depression due to the medical history of Crohn’s disease, our patient also possesses a significant family history of mental illness. When such risk factors are present, patient education and screening for psychiatric conditions are warranted before beginning treatment with infliximab. Furthermore, as our patient had a history of cannabis use before beginning infliximab treatment, it is also important to screen for substance use as it may further increase patients’ risks for the development of mood disorder and suicidal behavior. Patients should also be closely monitored during the initial phases of treatment to allow for early detection and treatment of neuropsychiatric symptoms that arise. Additionally, pretreatment of high-risk individuals who develop psychiatric adverse effects to infliximab with an atypical antipsychotic may improve its tolerability.^4^

## CONCLUDING SUMMARY

In summary, treatment with infliximab has likely contributed to the development of depression and suicidal behaviors in an adolescent with Crohn’s disease. Fluoxetine was effective for the treatment of infliximab-induced depression with suicidal thoughts and behaviors. Atypical antipsychotics have been recommended for refractory cases. In light of prior similar case reports, an association between treatment with infliximab and the onset of psychiatric symptoms is likely but requires further investigation. Patients treated with infliximab may benefit from screening and education about its rare, but serious, psychiatric effects, particularly if other risk factors such as personal or family history of mental illness are present.

## DISCLOSURE

The authors have no financial interest to declare in relation to the content of this article.
